# Functional Characterization of the Cardiac Ryanodine Receptor Pore-Forming Region

**DOI:** 10.1371/journal.pone.0066542

**Published:** 2013-06-12

**Authors:** Joanne Euden, Sammy A. Mason, Alan J. Williams

**Affiliations:** Institute of Molecular and Experimental Medicine, Cardiff University, Cardiff, United Kingdom; Dalhousie University, Canada

## Abstract

Ryanodine receptors are homotetrameric intracellular calcium release channels. The efficiency of these channels is underpinned by exceptional rates of cation translocation through the open channel and this is achieved at the expense of the high degree of selectivity characteristic of many other types of channel. Crystallization of prokaryotic potassium channels has provided insights into the structures and mechanisms responsible for ion selection and movement in these channels, however no equivalent structural detail is currently available for ryanodine receptors. Nevertheless both molecular modeling and cryo-electron microscopy have identified the probable pore-forming region (PFR) of the ryanodine receptor (RyR) and suggest that this region contains structural elements equivalent to those of the PFRs of potassium-selective channels. The aim of the current study was to establish if the isolated putative cardiac RyR (RyR2) PFR could form a functional ion channel. We have expressed and purified the RyR2 PFR and shown that function is retained following reconstitution into planar phospholipid bilayers. Our data provide the first direct experimental evidence to support the proposal that the conduction pathway of RyR2 is formed by structural elements equivalent to those of the potassium channel PFR.

## Introduction

The cardiac ryanodine receptor (RyR2) is located in the intracellular sarcoplasmic reticulum (SR) membrane network and is responsible for the regulated release of stored calcium from the SR lumen into the cytoplasm to initiate contraction in response to cell excitation [1].

**Figure 1:**
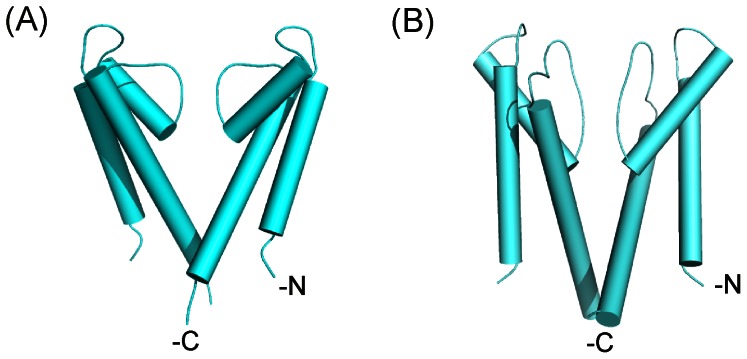
Comparison of the structural elements of KcsA and the RyR2 pore analogy model. (A) The pore forming region of KcsA and (B) our analogy model for the pore forming region of RyR2, constructed using KcsA as a template. Two monomers are shown for clarity as PyMOL cartoon representations (DeLano Scientific LLC, CA, USA).

Investigations using both molecular modeling [2], [3], [4] and cryo-electron microscopy [5], [6], [7] imply that the pore forming region (PFR) of RyR channel is composed of structural elements equivalent to those found in potassium channels and that these elements are likely to have a similar topology. In the absence of structural data at atomic resolution RyR PFR models provide us with useful frameworks that can be used to predict the involvement of domains and individual residues in the processes of ion translocation and gating (Figure 1).

Evidence to suggest that the PFR of RyRs could function as a separate entity can be found in the structural architecture of all potassium channels. The miniature viral potassium channel, Kcv, represents the pore module of all potassium channels and is fully functional when reconstituted into liposomes, displaying both cation selectivity and tetrameric stability [8]. Recent studies have also demonstrated that both the voltage-sensing domain (VSD) and pore domain (PD) in members of the voltage-gated ion channel (VGIC) family fold independently [9] and are capable of stand-alone function [10], [11]. These two domains are also interchangeable between family members [12]. To demonstrate structural modularity in this class of trans-membrane proteins, a recent study has shown that the pore domains of several voltage gated sodium channels are also able to fold independently and function as separate entities [13]. Although RyRs are not members of the VGIC family, the strong evidence suggesting structural similarities in the pore regions of these two types of channel indicates that the RyR PFR region may also be capable of independent expression and function. Support for this proposal has been provided by the demonstration that a region of the skeletal muscle isoform of RyR (RyR1) equivalent to the proposed PFR of RyR2 can be purified as a tetramer (9). Despite considerable circumstantial evidence, validation of this proposal requires the functional capabilities of the isolated putative RyR PFR to be assessed. In the current report we provide the first demonstration that the RyR2 PFR forms functional channels following incorporation into planar phospholipid bilayers.

## Materials and Methods

### Molecular Biology

Human RyR2-PFR cDNA was amplified by polymerase chain reaction (PCR) using the following primers that contained Nde1 and BamH1 restriction sites. The reverse primer also contained a 5′STOP codon. Forward 5′-GGAATTCCATATGTCAGTAACTCACAATGGCAAACAGC-3′ and Reverse 5′-CGGGATCCTCAATTTAGCTGGTCTTCATACTGTTTCCG-3′. PCR products were digested with Nde1 and BamH1 restriction endonucleases and ligated into Nde1/BamH1 digested pET19b vector (Novagen). Ligations were heat shocked into XL1 blue cells and clones were verified by restriction digestion and sequencing.

### Protein Expression

Following DNA sequencing, the *E.coli* derivative strain Rosetta (DE3) pLysS was transformed with the pET19b vector containing the correct cDNA. For protein expression cells were grown in Luria-Bertani (LB) broth at 37°C, with the addition of 100 µg/ml ampicillin, to an OD_600_ of 0.6–0.8. At this point the temperature was reduced to 20°C and protein expression was induced by the addition of 1 mM IPTG for 16 hours. Cells were harvested by centrifugation at 12,000× g for 20 minutes at 4°C. The supernatant, containing the soluble debris, was discarded and pellets were stored at −20°C until further use.

### Protein Purification

Cell pellets were defrosted at room temperature and resuspended in Tris buffer (50 mM Tris, 150 mM KCl, pH 8.0). Cells were lysed on ice by sonication (15 seconds on, 20 seconds off for five minutes) and cell membranes were collected by centrifugation at 100,000× g for 1 hour at 4°C.

Protein was solubilised for one hour at 4°C in Tris buffer containing 1% LDAO following dounce homogenization of the collected membranes. The solubilised sample was centrifuged at 25,000 g at 4°C for 20 minutes and supernatant was collected and loaded onto a disposable polypropylene column containing Ni-NTA beads pre-equilibrated with Tris buffer. 30 mM imidazole was added to the sample prior to loading to limit non-specific binding of other proteins. The column was washed extensively with Tris buffer containing 0.1% LDAO and 30 mM imidazole prior to elution of the bound protein with Tris buffer containing 300 mM imidazole and 0.1% LDAO. Eluted protein was collected straight into a 100-kDa MWCO centrifugal concentrator. Concentrated sample was filtered and loaded onto a HiLoad 16/60 superdex 200 column pre-equilibrated in Tris buffer containing 0.1% LDAO. Peak fractions were collected and used for biochemical and functional assays.

### Western Blotting

Samples were loaded onto a 10% polyacrylamide gel. Following electrophoresis, protein samples were transferred onto a polyvinilidine difluoride (PVDF) membrane (Millipore) overnight. Membranes were blocked in 5% non-fat milk in Tris-buffered saline (TBS) with 1% Tween-20 for one hour. Following three five-minute washes in TBS-Tween, membranes were incubated with mouse anti-His primary antibody (1∶5000 in 5% milk in TBS-Tween) for 90 minutes at room temperature. Following a second wash as above membranes were incubated in sheep anti-mouse-HRP secondary antibody for 90 minutes. Membranes were washed three times in TBS-Tween before ECL kit and film exposure.

### Reconstitution of hRyR2-PFR into sealed vesicles

5 mgs/ml L-α soy PC (Avanti) was solubilised in Tris buffer (50 mM Tris, 150 mM KCl, pH 8.0) before addition of 50 mM β-D- octyl glucopyranoside (OG). The sample was sonicated to clarity and purified hRyR2-PFR was added at a lipid to protein monomer ratio of 250∶1. Detergent was removed by the addition of 80 mg/ml washed SM2 biobeads (Bio-rad). The sample was left with gentle rotation for 4 h at room temperature before replacing biobeads with a second batch. The sample was left overnight at 4°C with gentle rotation. The reconstituted hRyR2-PFR sample was separated from the biobeads and flash frozen in liquid nitrogen in 100 µl aliquots and stored at −80°C until further use. Control vesicles were prepared as above but without the addition of protein.

### Single Channel Recording Experiments

Planar phospholipid bilayers consisting of phosphatidylethanolamine (Avanti Polar Lipids, Alabaster, AL) in decane (33 mg/ml) were formed across a 200 µm diameter hole in a polystyrene partition separating two fluid-filled chambers (*cis* and *trans*). The *trans* chamber was held at ground and the *cis* chamber was clamped at various holding potentials relative to ground using Ag-AgCl electrodes and bridges containing 2% agar in 3 M LiCl. Current flow through the bilayer was measured using an operational amplifier as a current-voltage converter (13). To assess the dependence of pore conductance on permeant ion concentration bilayers were formed in symmetrical solutions of 210 mM, 610 mM or 810 mM KCl with 20 mM HEPES, pH 7.4. Proteoliposomes containing purified PFR were added to the *cis* chamber following establishment of an osmotic gradient by the addition of 100 µl of 3 M KCl to the *cis* chamber. Following incorporation the *cis* chamber was perfused with the corresponding solution to give symmetrical conditions. The orientation of the PFR in proteoliposomes is unknown therefore it is impossible to distinguish whether the *cis* chamber corresponds to the cytosolic or luminal face of the pore. The permeability of Cl^−^ relative to K^+^ was determined by monitoring the reversal potential with 210 mM KCl in the *trans* chamber and 810 mM KCl in the *cis* chamber. To determine the relative permeability of the channel to K^+^ and Ca^2+^ the *cis* chamber was perfused with 210 mM CaCl_2_, adjusted to pH 7.4 with 20 mM Hepes. For investigations involving Neomycin, bilayers were formed in symmetrical 210 mM KCl and recorded at +60 mV prior to the addition of neomycin at either 10 µM or 100 µM to both the *cis* and *trans* chambers. All experiments were carried out at 22°C. Junction potentials were monitored and used to correct measurements of E_rev_ for calculation of relative cation-anion permeability using the Goldman-Hodgkin-Katz relationship.

### Calculation of open probability

The probability of the hRyR2-PFR being open was determined at a range of holding potentials. Preliminary inspection of the data established that bilayers routinely contained more than one channel and that open probability was very high across most of the range of potentials investigated. A cartoon representing a typical section of recording for a bilayer containing 3 hRyR2-PFR channels is shown as an inset in the corresponding figure for channel open probabilities. If a bilayer contains several (N), independently gating, identical channels, then the collective open probability distribution of these channels (Po_(N)_) is binomial [14], [15]. In these experiments Po_(N)_ was determined using QuB (version 1.5.0.19) which idealizes current fluctuations using the iterative hidden Markov model-based Baum-Welch expectation maximization algorithm. The total number of channels in the bilayer (N) was taken to be the maximum number of coincidentally open channels observed during the recording. Having determined Po_(N)_ and N, Po (the open probability of one of N channels) can be calculated as:
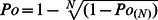



Given the high proportion of incomplete closing events in these experiments and that the majority of recordings rarely include coincident closing of more than one channel, this method provides a more accurate description of open probability than a conventional method in which open probability in bilayers containing more than one channel is determined by monitoring the time spent at each open level using a series of 50% thresholds [16].

## Results

### Expression and Purification of human hRyR2-PFR

hRyR2-PFR cDNA which encodes 209 residues (4758–4967) was cloned into pET19b and verified by DNA sequencing. Expression of the protein product was induced in *E.coli* cells to yield a 27.3-kDa protein with an N-terminal deca-His tag. A time course of isopropyl-β-D-thiogalactopyranoside (IPTG) induction showed adequate levels of protein expression at 3 hours post induction (data not shown). However, as the PFR construct constitutes only 4% of the total protein and functional status was initially unknown, standard protein expression was carried out at a reduced temperature of 20°C over 16 hours to encourage optimal protein folding. The PFR was purified as detailed in the methods section with an average yield of 1.5±0.5 mg per 1 L of bacterial culture. In accordance with results presented by Kang *et al.* [17], lauryldimethylamine oxide (LDAO) was found to solubilise RyR2-PFR with greater efficiency than 3-[(3-chloramidopropyl)di-methylammonio]-1-propanesulfonate (CHAPS), octyl-β-glucopyranoside (OG) and dodecyl-β-D-maltoside (DDM) (data not shown).

### Oligomeric Status of hRyR2-PFR

**Figure 2:**
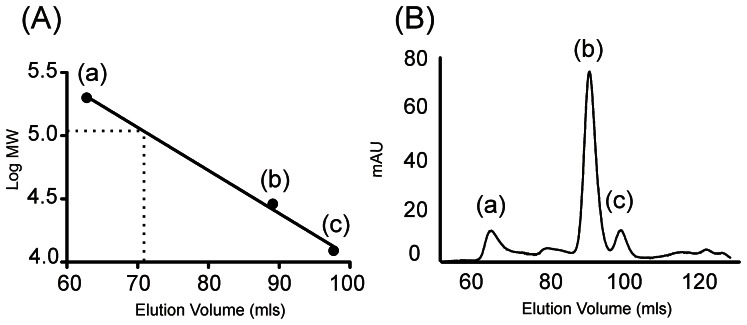
Analytical gel filtration of Calibration standards. (A) The logarithm of the molecular weight was plotted against elution volumes for three protein standards: (a) B-amylase (200 kDa), (b) carbonic anhydrase (29 kDa) and (c) cytochrome c (12.4 kDa) to create a calibration curve. The expected elution position of the hRyR2-PFR is shown as a dotted line. (B) Gel filtration trace of analytical standards.

**Figure 3:**
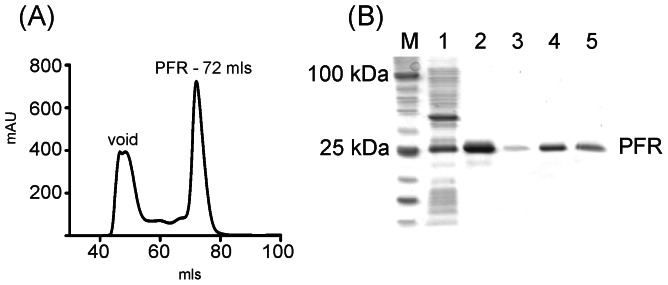
Purification of hRyR2-PFR. (A) Gel filtration trace of hRyR2-PFR showing void volume; and protein peak; (PFR) at 72 mls as predicted by analytical gel filtration. (B) Purification samples of hRyR2-PFR 1-Load, 2-affinity sample, 3-void, 4-main peak, 5-second affinity sample following GF.

A region of RyR1 equivalent to the proposed PFR of RyR2 has been expressed in bacteria. Following detergent solubilisation the purified protein was shown to be tetrameric by analytical gel filtration (GF) chromatography [17]. We have expressed the equivalent domain of RyR2 and have used the same analytical technique to demonstrate that the PFR from RyR2 also forms a folded, tetrameric protein when cloned into a vector with an N-terminal His-tag and expressed in Rosetta cells. A plot of the log molecular weights of protein standards against elution volume is shown in Figure 2A, highlighting the expected elution position of hRyR2-PFR, together with the analytical gel filtration trace (Figure 2B). The gel filtration profile of the hRyR2-PFR in Figure 3A is a narrow, sharp peak indicating a monodisperse sample with an elution volume of 72 mls, which is in good agreement with the expected elution volume of 71 mls for a protein with a theoretical mass of 109 kDa for a tetramer. Calculations for an associated detergent micelle of LDAO (17 kDa) only shift the expected position by 2 mls to a predicted elution volume of 69 mls. As the protein standards were soluble proteins, the predicted elution positions will not be wholly accurate for a membrane protein. Nonetheless, the observed GF profile is in good agreement with that predicted. The protein could be purified to homogeneity as indicated by the purification samples in Figure 3B. DDM gave a similar purification to that observed with LDAO. CHAPS appeared to be the worst candidate, giving rise to multiple protein peaks corresponding to higher molecular weight aggregates and OG retained most protein in the void volume (data not shown).

### Single Channel Activity of hRyR2-PFR - General Observations

**Figure 4:**
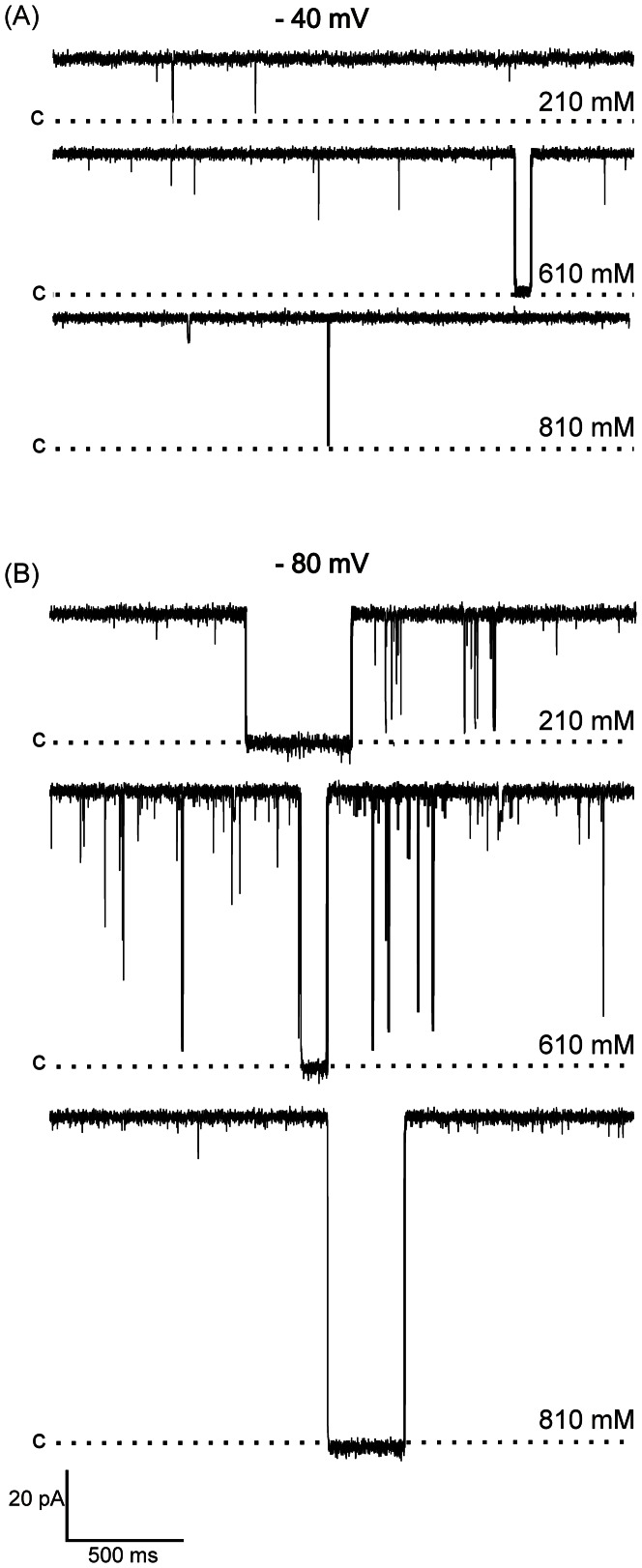
Single channel traces showing high P_o_ of hRyR-PFR2 at a range of ionic activities. (A) shows recordings at −40 mV with closings shown as downward deflections. P_o_ is extremely high with very rare closing events. Closings are often too short to be fully resolved. (B) shows recordings at −80 mV. Closings occur more frequently and dwell times within closed states are much longer. Closed levels are denoted by C with the hashed line representing the closed level.

**Figure 5:**
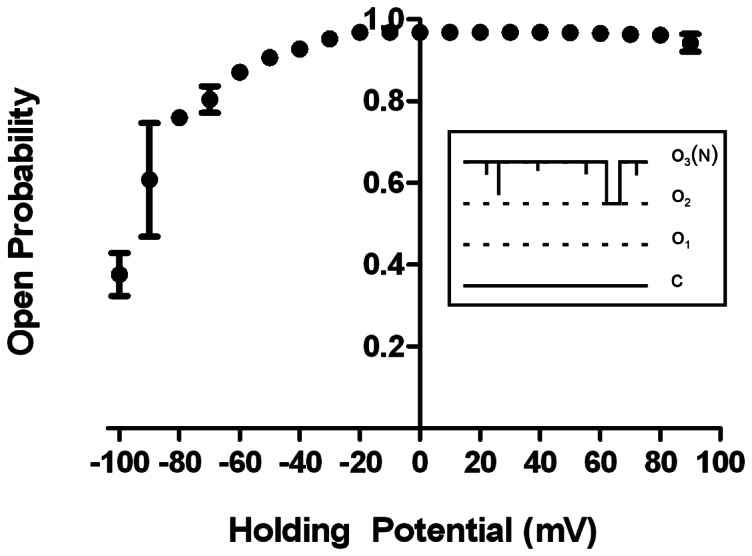
Open probabilities of hRyR2-PFR. A plot of open probability (Po) plotted against voltage is shown in symmetrical 210 mM KCl. All points are mean values ±SD derived from three or more experiments. Error bars, where not visible, are within the symbol. The cartoon inset shows a stylized representation of a typical section of recording from a bilayer containing 3 hRyR2-PFR channels at −80 mV. The collective open probability of these channels (Po_(N)_) was determined as the time spent at level N (level 3 in this example), as a proportion of the total time of the recording using QuB, as described in Materials and Methods.

Our biochemical results provide strong indications that the hRyR2-PFR is a folded tetrameric protein. However, the primary aim of this study was to establish if the proposed PFR retains functional integrity as a separately expressed domain. To test this we fused liposome-reconstituted purified hRyR2-PFR with planar phospholipid bilayers and monitored channel gating and ion handling under voltage clamp conditions. Fusion events routinely led to the incorporation of multiple copies of one form of channel (this channel was not seen following the fusion of control material – see methods). When monitored in symmetrical 210 mM KCl at holding potentials of ±10 to ±40 mV these channels were characterized by an extremely high open probability (P_o_) with only very rare and incompletely resolved closing events. However the gating of the PFR does show an appreciable level of voltage dependence with a reduction in channel P_o_ as holding potential is taken to high negative voltages (Figure 4). At holding potentials of −80 mV, or more negative, successive shutdowns of channels is observed although channels can be reopened with a reversal in holding potential. At high negative potentials P_o_ remains high, however dwell times in the closed state are markedly increased (Figure 4B). In contrast, no observable difference in P_o_ was observed at high positive holding potentials with only very rare, short-lived, closings across the entire voltage range (Figure 5).

The ryanodine receptor is so named because the full-length channel contains a high-affinity binding site for this plant alkaloid. Interaction of ryanodine with the channel results in characteristic changes in both channel gating and ion handling. We tested if the ryanodine binding site was preserved in the hRyR2-PFR by adding 4 µM ryanodine to the solutions on both sides of bilayers containing these channels. The experiment was repeated 6 times with activity monitored, in each case, for at least 10 minutes, however we observed no ryanodine-dependent alteration in hRyR2-PFR conductance or gating (data not shown).

### Ion translocation and discrimination in the hRyR2-PFR

**Figure 6:**
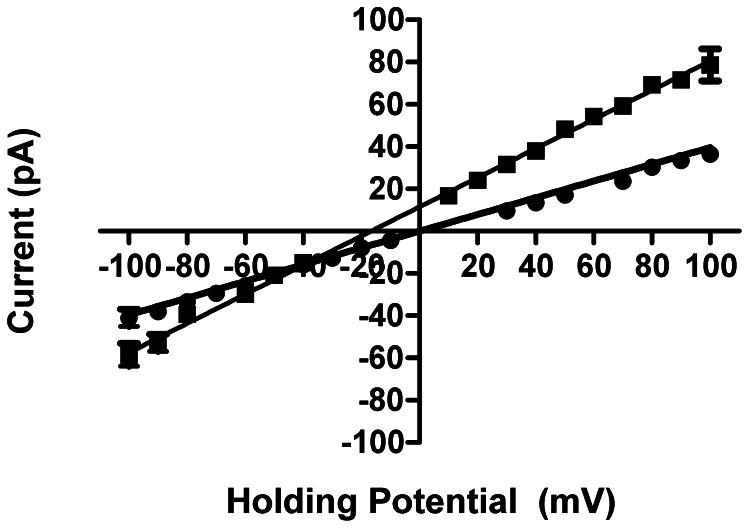
Cation-anion discrimination in hRyR2-PFR. Current-voltage plots showing single channel current amplitude of PFR in symmetric 210 mM KCl (•) and asymmetric KCl (210 mM *trans*; 810 mM *cis*) (▪). The linear regression plot through the asymmetric K^+^ data indicates a reversal potential of −17 mV. This indicates that the PFR alone has lost some ability to distinguish between cations and anions. All points are mean values ±SE derived from five or more experiments. Error bars, where not seen, are within the symbol.

The full-length RyR2 channel is impermeable to anions and shows a degree of discrimination between divalent and monovalent cations (pX^2+^/pY^+^≈6.5) [18]. Cation translocation in the channel is characterized by high unitary conductance that saturates with increasing permeant ion activity [19]. In symmetrical 210 mM KCl single hRyR2-PFR current varies linearly with holding potential (Figure 6) with a slope conductance of 395 ±8 pS. This is significantly lower than the slope conductance of the full-length RyR2 under these conditions (806±4 pS). The relative permeability of the hRyR2-PFR to monovalent cations and anions was determined by monitoring the reversal potential (E_rev_) with a KCl gradient across the bilayer (210 mM trans∶810 mM cis). The resulting current-voltage relationship is linear with an E_rev_ of −15.3 mV (Figure 6) indicating a relative permeability of K^+^ over Cl^−^ (calculated from the Goldman-Hodgkin-Katz equation) of 3.03. Therefore while the PFR retains some ability to discriminate between cations and anions this property is reduced significantly compared to the situation in the full-length channel and monitored hRyR2-PFR currents will have both a cation and an anion component.

**Figure 7:**
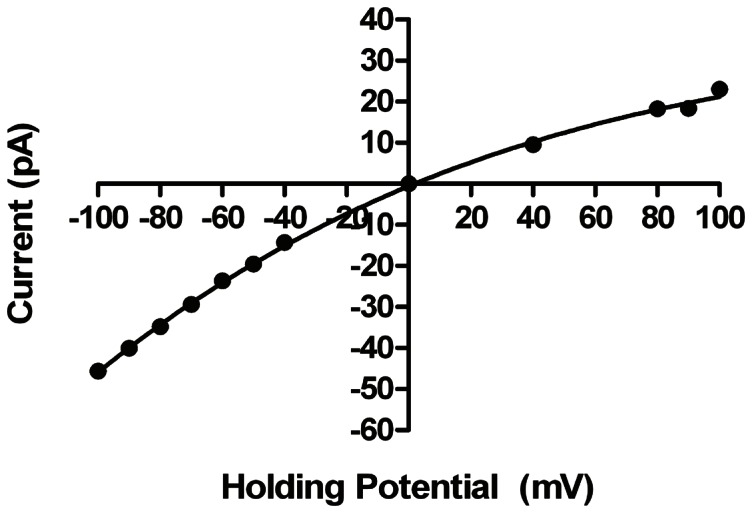
Monovalent-Divalent discrimination in hRyR2-PFR. Current-voltage plot showing the response of hRyR2-pFR in asymmetric conditions of 210 mM KCl (*trans*) and 210 mM CaCl_2_ (*cis*). The fitted curve has no mathematical significance. All points are mean values ±SE derived from four or more experiments. Error bars, where not visible, are within the symbol.

To assess whether the ability of the hRyR2-PFR to discriminate between monovalent and divalent cations is comparable to that of the full-length hRyR2, current-voltage relationships were obtained in 210 mM CaCl_2_ (*cis*) and 210 mM KCl (*trans*). The data in Figure 7 demonstrate that in these conditions unitary current in the hRyR2-PFR reverses at a holding potential of 0 mV, indicating that, unlike full-length RyR2, the hRyR2-PFR is unable to discriminate between Ca^2+^ and K^+^. With these ionic conditions, although hRyR2-PFR is to some extent permeable to Cl^−^, the slope conductances monitored at high positive and high negative holding potentials will predominantly reflect current carried, respectively by, Ca^2+^ and K^+^. The lower conductance of Ca^2+^ indicates that although Ca^2+^ and K^+^ are equally permeant, as is the case in the full-length RyR2, the rate of K^+^ translocation in the hRyR2-PFR exceeds that of Ca^2+^.

**Figure 8:**
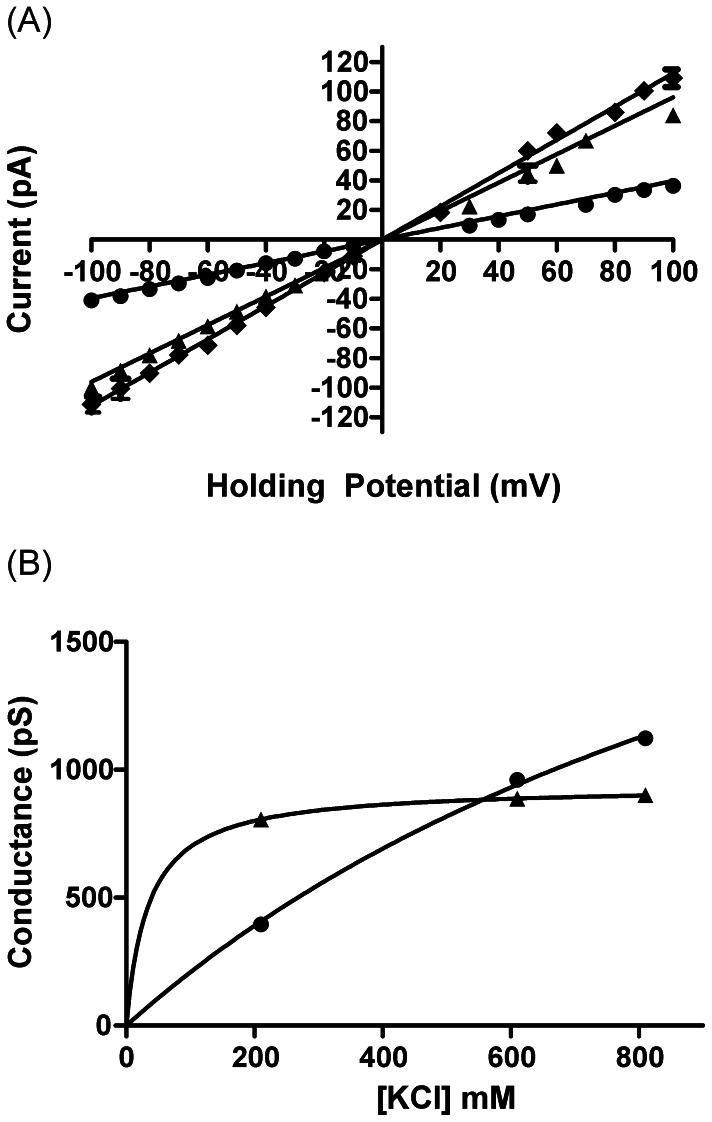
Single channel conductance of hRyR2-PFR. (A) Current voltage plots of hRyR2-PFR in symmetrical 210 mM (•), 610 mM (▴), and 810 mM (⧫) KCl. Solid lines are derived from linear regression to give the slope conductance. All points are mean values ±SE derived from four or more experiments. Error bars, where not visible, are within the symbol. (B) shows the change in slope conductance with an increase in K^+^ concentration for WT hRyR2 (▴) and the PFR (•). Lines are nonlinear regression derived from a single-site binding hyperbola. All points are mean values ±SE derived from four or more experiments. Error bars are within the symbol.

Single hRyR2-PFR conductance varies with the concentration of permeant ions. Current-voltage relationships in symmetrical 210, 610 and 810 mM KCl are shown in Figure 8A with slope conductances of 395 ±8 pS, 960±9 pS and 1123±13 pS respectively. The relationship between PFR slope conductance and KCl concentration is shown in Figure 8B where these data are plotted alongside equivalent data for the full-length RyR2 channel. As noted above, hRyR2-PFR conductance in symmetrical 210 mM KCl is significantly lower than that of the full-length RyR2. In symmetrical 610 mM KCl conductance of both channels is similar and in symmetrical 810 mM KCl the conductance of the PFR is significantly greater than that of full-length RyR2. Whereas K^+^ conductance in full-length channel saturates at concentrations in excess of 200 mM, conductance in hRyR2-PFR shows little sign of saturation under the conditions tested here.

The data presented in Figures 6–8 demonstrate that the characteristics of ion discrimination and translocation in the hRyR2-PFR are somewhat different from those of the full-length RyR2 channel. The ability to exclude anions is markedly reduced, discrimination between divalent and monovalent cations is lost, the affinity for permeant ions is decreased, while the maximum rate of ion translocation through the pore is increased.

### Influence of RyR2 blockers on ion handling in the hRyR2-PFR

Cation translocation through the full-length RyR2 channel can be modified by a variety of blocking molecules [20] [21]. We have examined the influence of representative blockers of the full-length RyR2 on hRyR2-PFR. Tetrabutylammonium (TBA) and tetrapentylammonium (TPeA) are organic cations that interact with residues in the cytosolic vestibule of the full-length RyR2 channel [22]. When bound these molecules partially occlude the conduction pathway producing well-resolved blocking events with characteristic residual currents. The influence of TBA and TPeA on ion conduction through the hRyR2-PFR was investigated by adding 100 µM of these potential blockers to both *cis* and *trans* chambers: neither cation produced any alteration in hRyR2-PFR function (data not shown).

**Figure 9:**
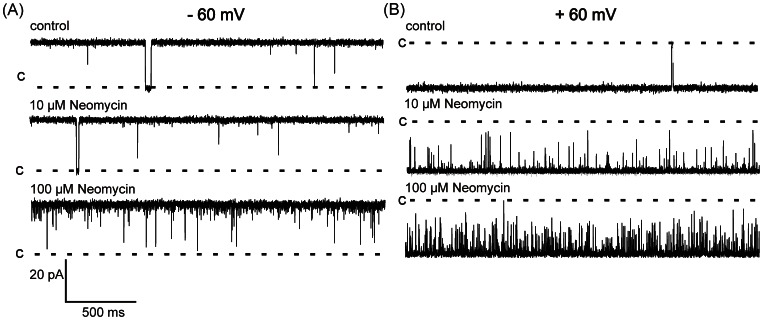
Single Channel traces showing effects of neomycin on Po of hRyR2-PFR in 210 mM KCl. The indicated concentrations of neomycin were present in both the *cis* and *trans* chambers. (A) shows recordings at −60 mV with closings shown as downward deflections. Po is extremely high with very rare closings. Upon the addition of neomycin blocking become more apparent although events are often too brief to be resolved. (B) shows recordings at +60 mV where the addition of neomycin has a more noticeable effect on Po. Blocking events are shown as upwards defections.

**Figure 10:**
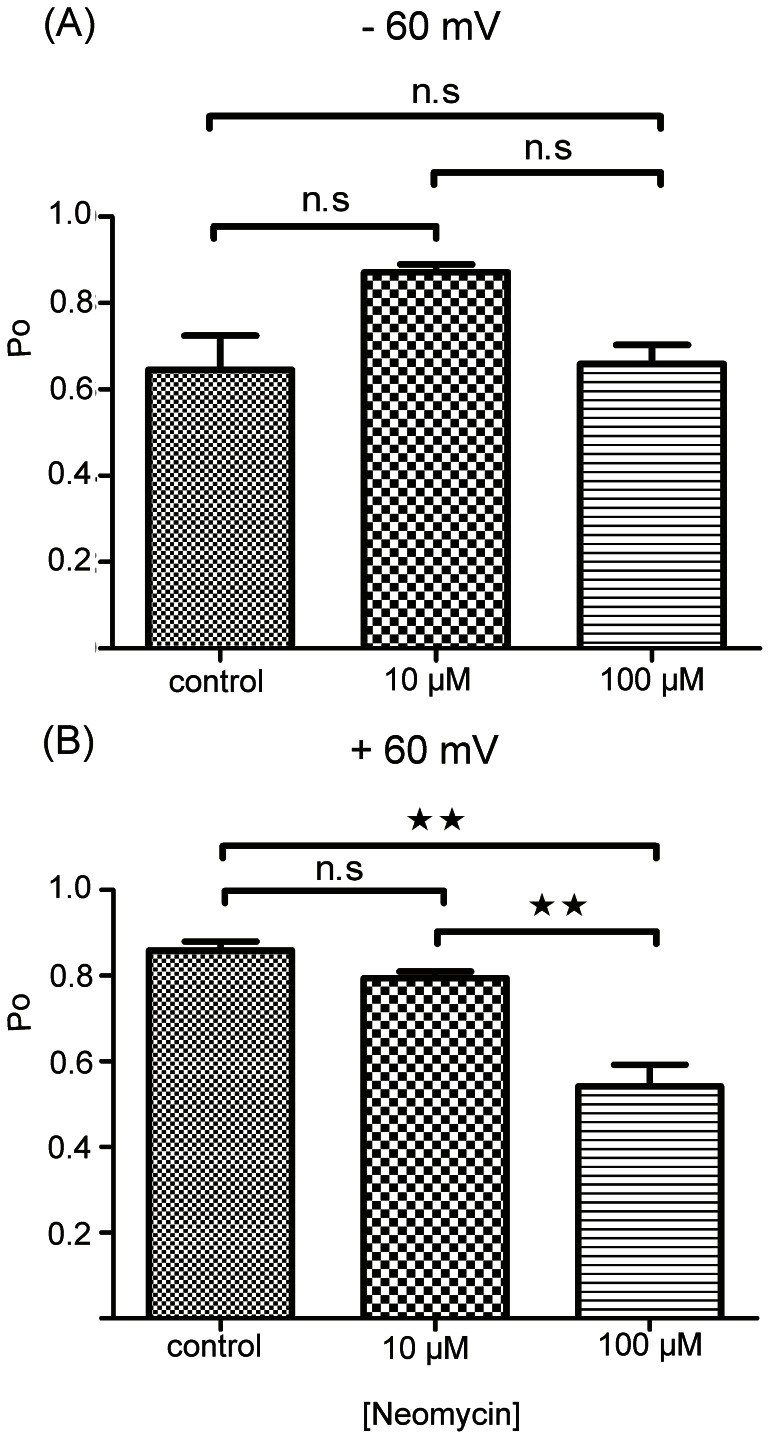
Effect of Neomycin on Channel Open Probability. (A) shows Po of hRyR2-PFR at −60 mV with neomycin added to both sides of the channel. (B) shows Po of hRyR2-PFR at +60 mV under the same conditions. Data are plotted as mean ±S.E.M for between three and six channels. Where differences between the means reach statistical significance, these are indicated by asterisks (**P<0.005).

Neomycin is a polycationic, aminoglycoside antibiotic that is a potent blocker of the full-length RyR2 channel [23]. Unlike other pore blockers of full-length RyR2, neomycin blocks from both the cytosolic and luminal side of the channel, partially occluding the pore and producing clearly resolved blocking events with characteristic residual currents. We investigated potential interactions between neomycin and the hRyR2-PFR by adding increasing concentrations of the polycation to both the *cis* and *trans* chambers and monitoring current fluctuations at both positive and negative holding potentials. As demonstrated in Figure 9 neomycin produces very brief, incompletely resolved, transitions from the open state of the hRyR2-PFR. These events were more frequent at positive than negative holding potentials and their probability of occurrence increased as the concentration of neomycin was raised. The influence of neomycin on hRyR2-PFR open probability is quantified in Figure 10. No significant reduction in Po was observed at −60 mV but Po was significantly reduced in the presence of 100 µM neomycin at +60 mV. The logical interpretation of these observations is that neomycin has preferential access to the open hRyR2-PFR from the *cis* chamber and, when present, can block permeant ion translocation. The duration of individual blocking events is very short.

As a byproduct, the asymmetric effect of neomycin on the hRyR2-PFR provides us with information about the way in which the channel incorporates into the bilayer. The markedly greater influence of neomycin at positive holding potentials was observed in all experiments indicating that the hRyR2-PFR incorporates into the bilayer in a preferred orientation.

## Discussion

Sequence analysis, cryo-EM and molecular modeling [3], [7], [24] indicate that the PFR of the RyR channel likely contains structural elements equivalent to those found in potassium channels, and correlations between experimental data and molecular dynamics simulations demonstrate that the crystal structure of KcsA provides an excellent template for the PFR of RyR2. Single channel studies of full-length RyR in which mutations have been inserted in the proposed ion conduction pathway and pore blocking experiments support this model [4], [18], [24], [25], [26], [27], [28]. However, the detailed architecture and mechanisms of ion handling of the putative PFR remain to be established. The observation by Kang *et al.*,[17] that the proposed PFR of RyR1 could be expressed and purified as a tetrameric complex, provided an opportunity to progress our knowledge beyond a hypothesis and characterize the structure and function of this domain, providing direct evidence that this region does form a gated ion-conduction pathway with stand-alone function. In the present study we have cloned the proposed PFR of hRyR2 into a bacterial expression vector, isolated it following detergent solubilisation and reconstituted it into planar phospholipid bilayers to characterize function.

Following a two-stage purification process our results, in accordance with those of Kang *et al.*,[17] show that it is possible to purify the hRyR2 PFR as a stable tetrameric complex in large amounts.

Our results establish that hRyR2-PFR forms a functional ion channel when reconstituted into planar phospholipid bilayers. However, deletion of 96% of the full-length protein has a significant effect on both channel gating and ion handling. Gating of the full-length RyR2 channel is regulated by many physiological and pharmacological ligands [29], [30]. While the sites of interaction of these ligands remain to be identified, the majority must bind on or in the bulky cytoplasmic domain of the channel. A recent study by Ramachandran *et al* also shows that contacts made between the inner helix of the putative PFR and a proposed S4–5 linker helix of the full-length RyR1 channel play a pivotal role in controlling gating, with mutations in the linker stabilizing either a closed or an open conformation of this channel [4].

In the absence of the cytoplasmic domain, the remaining trans-membrane helices, or any other regulatory mechanism, the hRyR2-PFR has the potential to exist in three conformations: permanently open, permanently closed or capable of undergoing random transitions between open and closed conformations. In reality, under conditions where the full-length channel would have a low open probability [31], we see a channel that is predominantly open with brief, often poorly resolved, closing events. Gating of the full-length RyR2 channel is altered dramatically following the high-affinity interaction of ryanodine. Although the location of the ryanodine binding site remains to be established, a considerable body of indirect evidence indicates that it may be within the PFR of the channel [25], however exposure of the hRyR2-PFR to ryanodine produced no change in either the gating or ion handling properties of the channel. An absence of effect could mean either a) that the binding site for ryanodine is not within the PFR, b) that the site, if present, differs from that in the full-length channel and prevents binding or c) that binding of ryanodine to the site does not result in conformational alterations that modify hRyR2-PFR function.

The ion-handling properties of the hRyR2-PFR differ considerably from those of the full-length channel. These differences are not entirely unexpected and most likely arise from disparities in the structure of the conduction pathways of the two channels, indicating that other domains of the full-length RyR2 influence ion handling in the PFR. As highlighted in our original description of the RyR2 PFR model [2], these influences could be direct (by altering the electrostatic profile experienced by ions approaching the PFR [32]), or indirect (by influencing the arrangement of domains and residues within the PFR [4]). Investigations in both RyR and other channel types suggest that significant changes in ion handling can result from only minor differences in structure.

Unlike full-length RyR2 [19] discrimination between cations and anions in the isolated PFR is almost completely lost. While the structural basis for the ability of RyR2 to discriminate between cations and anions is yet to be established, data obtained in other systems may be relevant. Neutralization of a ring of acidic residues at the entrance to the 5-HT_3_ receptor PFR renders this cation-selective channel equally permeable to monovalent cations and anions [33]. Alternatively, residue substitutions that increase the minimum pore radius of glycine receptor channels can have very significant effects on their ability to discriminate between anions and cations and it has been proposed that counter-cations can be translocated through these channels in neutral ion pairs, with their permeability dependent upon the hydrated diameter of the ion pair relative to the minimum diameter of the pore [34]. The increased permeability of hRyR2-PFR to anions could arise from a structural modification that results in either a redistribution of acidic residues at the cytosolic (or luminal) entrance to the pore or in an increase in the minimum radius of the pore.

In addition to the marked loss of cation-anion discrimination, the relationship between unitary conductance and permeant ion concentration of the isolated hRyR2-PFR differs from that seen in the full-length channel. In full-length RyR2 the relationship between unitary conductance and potassium activity can be described by a simple saturation scheme, with conductance reaching a maximum at activities in excess of 200 mM. In the hRyR2-PFR unitary conductance is lower than that of the full-length channel at KCl concentrations below 600 mM and, although conductance does not rise linearly with increasing ionic concentration, conductance shows little sign of saturation at 800 mM KCl.

In theory the very high rates of cation conductance typical of the full-length RyR2 could be achieved if the physical characteristics of the channel's PFR a) maximize rates of ion delivery (entrances bearing a significant net negative charge and large water filled vestibules either side of a selectivity filter) and b) a selectivity filter with dimensions (short and relatively wide) that would maximize rates of ion translocation. Both functional [35], [36] and modeling investigations [2] indicate that the RyR2 PFR is likely to have both of these structural specializations. What structural modifications might underlie the conductance characteristics displayed by the isolated hRyR2-PFR? A relationship between single channel conductance and activity very similar to that observed for the hRyR2-PFR was seen following the neutralization of acidic residues identified at the luminal entrance to the selectivity filter in the RyR2 PFR model. The combined replacement of E4832 and D4833 with alanines yielded a channel that, although it retained the ability to discriminate between cations and anions, displayed reduced conductance at sub-saturating potassium activities with little indication of saturation at activities of 700 mM. Comparisons of the electric fields of, and molecular dynamics simulations in, the WT and ED4832AA RyR2 PFR models, indicated that the observed changes in conductance could result from differences in the electric field (at the luminal face of the pore and in the selectivity filter) and differences in the stability of residues within the selectivity filters of the two structures [32]. Equivalent discrepancies in structure resulting from the absence of the trans-membrane and cytosolic domains present in the full-length channel likely underlie the modified conductance properties of the isolated hRyR2-PFR.

This conclusion is supported by our observations with well-characterised blockers of the full-length RyR2 channel. TBA and TPeA are effective blockers of full-length RyR2 at µM concentrations but produced no discernable block in the hRyR2-PFR. In contrast neomycin, a much higher affinity blocker of full-length RyR2 (effective in nM range), does interact with the hRyR2-PFR to produce a degree of block. However the concentrations required to produce block are much higher than those required in the full-length channel and the morphology of the resulting events differ considerably from those produced in intact RyR2. Again these observations indicate that the cytosolic domain and other trans-membrane domains have significant roles in determining the structure of the PFR in the full-length RyR2 channel.

While both experimental and theoretical considerations suggest that the conduction pathways of RyR channels are likely to be formed by structural elements equivalent to those found in potassium channels, to date no direct demonstration of this has been presented. To provide this evidence we have expressed 209 residues from the C-terminus of the human RyR2 channel in bacteria, isolated the protein as a tetramer and characterized its function. Reconstitution of the hRyR2-PFR into planar bilayers establishes that this region of the molecule can function as a gated ion channel. Differences in gating and the properties of ion handling between the hRyR2-PFR and the full-length RyR2 channel indicate that other domains of the full-length molecule contribute, either directly or indirectly, to the detailed structure of the PFR and hence the mechanisms governing function in the intact tetramer.
